# Interviewing in virtual environments: Towards understanding the impact of rapport-building behaviours and retrieval context on eyewitness memory

**DOI:** 10.3758/s13421-022-01362-7

**Published:** 2022-10-17

**Authors:** Coral Dando, Donna A. Taylor, Alessandra Caso, Zacharia Nahouli, Charlotte Adam

**Affiliations:** 1 University of Westminster, Psychology, London, UK; 2 University of Derby, Psychology, Derby, UK

**Keywords:** Eyewitnesses, Episodic memory, Virtual Environment, Rapport, Cognitive Resources

## Abstract

Given the complexities of episodic memory and necessarily social nature of in-person face-to-face interviews, theoretical and evidence-based techniques for collecting episodic information from witnesses, victims, and survivors champion rapport-building. Rapport is believed to reduce some of the social demands of recalling an experienced event in an interview context, potentially increasing cognitive capacity for remembering. Cognitive and social benefits have also emerged in remote interview contexts with reduced anxiety and social pressure contributing to improved performance. Here, we investigated episodic memory in mock-eyewitness interviews conducted in virtual environments (VE) and in-person face-to-face (FtF), where rapport-building behaviours were either present or absent. Main effects revealed when rapport was present and where interviews were conducted in a VE participants recalled more correct event information, made fewer errors and were more accurate. Moreover, participants in the VE plus rapport-building present condition outperformed participants in all other conditions. Feedback indicated both rapport and environment were important for reducing the social demands of a recall interview, towards supporting effortful remembering. Our results add to the emerging literature on the utility of virtual environments as interview spaces and lend further support to the importance of prosocial behaviours in applied contexts.

## Introduction

The information provided by witnesses, victims and survivors^1^ is fundamental to the investigation of crime and prosecution of perpetrators. When providing an account, witnesses are asked to reconstruct and recount personally experienced episodes that occurred in a particular temporal and spatial context (Tulving, 1993). Episodic memory is a complex cognition which conceptually comprises several distinct components—what, when, and where—accompanied by a feeling of reexperiencing (Conway & Pleydell-Pearce, 2000; Tulving, 1993). Witness information is usually collected during an interview, typically conducted in person, face-to-face by a police officer or similar professional investigator. Given the complexities of episodic memory and necessarily social nature of face-to-face interview contexts, many theoretical and evidence-based witness interview techniques champion rapport-building as a technique to support the development of a positive interaction and help manage the power imbalance between the professional interviewer and witness (e.g., Ministry of Justice, 2022—Cognitive Interview; National Institute of Child Health and Human Development Protocol; PEACE Model).

Rapport-building, also referred to as prosocial behaviour or supportive/attentive behaviour, is believed to be important for relieving some of the social demands of an interview (e.g., Kieckhaefer et al., 2014; Nahouli et al., 2021; Roberts et al., 2004; Webster et al., 2021), potentially increasing cognitive capacity for remembering (Dando et al., 2016; Fisher & Gieselman, 1992; Milne & Bull, 2016; Nahouil et al., 2021; Webster et al., 2021). Comfortable witnesses may well be better placed to devote finite cognitive resources to complex cognitions, here recalling episodic experiences (e.g., Fiske & Taylor, 2013; Frith & Frith, 2012; Gallese et al., 2004). It seems sensible to suggest that socially comfortable witnesses will be “better” witnesses; however, the experimental rapport-building literature in this regard is limited. There is no widely agreed definition of rapport, and as a recent review has revealed, the experimental literature has tended to emphasize verbal rapport-building (see Gabbert et al., 2021). Presumably, because verbal behaviour is more straightforward to operationalise, control, and analyse than nonverbal behaviour. Furthermore, research findings are very mixed, likely because rapport is often subjectively described, for example as a bond, a connection to another, and a communicative alliance, and rapport behaviours are variously operationalised, both theoretically and empirically. Further, different clusters of rapport behaviours are applied, and rapport is not measured consistently across studies.

Rapport-building is not a prescriptive process and is thought to comprise a wide range of

physical and/or verbal behaviours. In investigative interview contexts rapport-building behaviours are generally thought to be verbal, facial, and physical in nature. Examples include active listening (smiling, nodding, & uh-huh), immediacy behaviours (eye contact & leaning forward), self-disclosure (Abbe & Brandon, 2014), tone of voice and empathy (e.g., Baker-Eck et al., 2020; Dando & Oxburgh, 2016; Griffiths & Rachlew, 2018; Jakobsen, 2021), and personalising the interview (Fisher & Geiselman, 1992). Indeed, professional training materials and guidance for conducting interviews with witnesses emphasize and describe various rapport-building behaviours. For example, the Cognitive Interview technique (Fisher & Geilselman, 1992), UK College of Policing (2021) and the Norwegian Police College (Fahsing & Rachlew, 2009; Rachlew & Fahsing, 2015) all suggest several verbal behaviours, including personalising the interview process by “introducing yourself,” interacting meaningfully with the witness “making it feel like a two-way conversation,” and making the conversation “natural and simple.” Less guidance is offered on nonverbal behaviour, simply that head-on face-to-face interactions should be avoided and that interviews should be calm, and interviewers should respect witnesses’ personal space. The recent Ministry of Justice (2022) Achieving Best Evidence guidance suggests, for example, beginning by conversing about neutral topics using easily answered, predominantly open questions, and using supportive behaviours such as active listening (Achieving Best Evidence guidance relates to England and Wales). The PEACE investigative interviewing model (relevant to England and Wales), the PRICE model (relevant to Scotland), and the Norwegian KREATIV national investigative interviewing training program emphasise the use of neutral open questions in establishing rapport in terms of supporting the witness to answer positively to create a positive mood. The Cognitive Interview (Fisher & Geiselman, 1992) suggests rapport can be built and maintained by personalising the interview process, interacting meaningfully with the witness, being attentive and transferring control of the interview from the interviewer to the witness (Memon & Higham, 1999).

Despite varying operationalisations of rapport-building, there is consensus that *some* rapport-building behaviour is better than none for positive outcomes (e.g., College of Policing, 2018; Gabbert et al., 2021; Ministry of Justice, 2022; Milne & Bull, 1999; Nahouli et al., 2021; Nash et al., 2014; Nash et al., 2016; Walsh & Bull, 2012), although experimental research findings are mixed. Some research indicates witnesses can provide more complete and accurate accounts when rapport-building behaviours are present (e.g., R. Collins et al., 2002; Holmberg & Madsen, 2014; Nahouli et al., 2021; Nash et al., 2016; Vallano & Shreiber Compo, 2011; Novotny et al., 2021), but this is not always the case (see Kieckhaefer et al., 2014; Sauerland et al., 2018). For example, verbal rapport-building (unaccompanied by appropriate behaviours) has been found to increase information yield (e.g., Novotny et al., 2021), whereas some have reported that verbal behaviour alone is less effective (e.g., Nahouli et al., 2021). In a similar vein, extensive rapport-building (comprising both verbal and behavioural techniques), has been reported to improve recall performance (e.g., Collins et al., 2002; Kieckhaefer et al., 2014; Nahouli et al., 2021), while others have reported no positive impact (e.g., Meissner et al., 2015; Sauerland et al., 2018). A recent review of the use of rapport by professionals during interviews with witnesses and suspected offenders/persons of interest has indicated that some form of rapport does improve outcomes in the majority of cases reviewed (Gabbert et al., 2021).

Most experimental rapport research has employed traditional in-person face-to-face interview paradigms (although see Drolet & Morris, 2000; Nunan et al., 2020). Yet the COVID-19 public health emergency has forced organisations to consider virtual or remote interview solutions, bringing into sharp focus just how little remote interviewing research has been conducted. Rapport-building in remote information gathering interviews has received little attention versus traditional in-person face-to-face contexts despite increasing digital adoption that is changing the way that organisations do business, including police and government bodies (e.g., national crime agency). Here, we investigate witness memory in remote virtual environments and explore the impact of a cluster of basic rapport behaviours that are drawn from contemporary best practice guidance, and which are theoretically and empirically supported (see below).

### Virtual environments as interview spaces

Virtual environments (VEs) are immersive computer simulations with a high degree of realism (Loomis et al., 1999; Taylor & Dando, 2018; Witmer & Singer, 1998) that offer opportunities as remote witness interview spaces. VEs render visual, auditory, and haptic information within milliseconds, bringing about realistic behaviour because the environment “feels” real, thus leveraging behavioural responses to environmental changes and challenges (e.g., Slater, 2009; Gonzalez-Franco & Lanier, 2017). VEs can be quickly and remotely created and managed using widely accessible portable computer and smartphone technology. In VEs, people communicate as *avatars* (see Ahn et al., 2013), which allows them to interact realistically albeit in the absence of physical co-presence (Baccon et al., 2019; Kang & Watt, 2013). The extant literature on avatar-to-avatar communication highlights potential cognitive and social benefits, suggesting interviews in VEs may be as efficient as face-to-face in-person witness interviews in some instances.

Non-investigative interviewing research, that is research concerned with interviews conducted for reasons other than the investigation of crime, reveals improved outcomes and better interviewer/interviewee experiences. Examples include enhanced disclosure of information (e.g., Baccon et al., 2019; Joinson, 2001; Suler, 2004) and reduced performance anxiety (e.g., Omarzu, 2000; Rubin, 1975). Interviewees and interviewers have also reported less social pressure (Baccon et al., 2019; Herrera et al., 2018) and increased confidence (e.g., Salmon et al., 2010). More recently, online simulation training using avatars was found to improve the quality of clinical psychologists’ interviewing (Haginoya et al., 2021; Pompedda et al., 2022). Avatar-to-avatar nonverbal communication has also been found to increase co-operation, lowering the need for additional verbal interactions to achieve efficient outcomes that require social co-operation (Greiner et al., 2014). Similarly, improved interpersonal trust and more impactful nonverbal behaviours have been reported when communicating avatar-to-avatar versus other communication contexts (e.g., Bente et al., 2008; Roth et al., 2017; Segal et al., 2022).

As far as we are aware the only published research conducted in a VE with mock witnesses is that by Taylor and Dando (2018) who found some advantages of gathering witness information in VEs akin to some of the benefits reported in the non-investigative literature. Episodic performance improved, with a significant reduction in errors, mirroring positive findings reported by others where memory and related cognitions were investigated in a VE (Bailenson et al., 2008; Saidel-Goley et al., 2012). Taylor and Dando also reported that the VE was well received. Interviewee experiences were extremely positive, including participants feeling more comfortable explaining when they did not know the answer or could not remember and enhanced concentration, which may have contributed to improved performance.

This pattern of results alongside findings from the wider literature suggest improved performance in a VE may emanate from a combination of reduced social demand (typically experienced in human-to-human interactions) and an absence of external stimuli, potentially reducing a dual cognitive task (episodic remembering and social monitoring) to a single task (remembering). Avatars represent the presence of another, offering social and communication benefits but without being physically co-present, perhaps limiting the potentially confounding influence of others on cognition, including memory (e.g., Brewer & Feinstein, 1999; Fiske, Lin, & Neuberg, 2018; Macrae & Bodenhausen, 2000; Maddox et al., 2008). Given that episodic recall is a demanding cognitive task, requiring a subjective sense of time (mental time travel), and a connection to the self and autonoetic consciousness, it is reasonable to suggest that an absence of external stimuli and better managed social demand will support improved performance.

### Remote rapport-building

Numerous positive benefits of rapport-building in remote avatar-to-avatar communication have been reported, including inducing strong feelings of positivity (Rehm et al., 2016), improved social engagement (Peyroux & Frank, 2014), and increased self-disclosure (Lee & Dryjanska, 2019; Pickard et al., 2016). However, as far as we are aware, no experimental rapport-building research has been conducted in VE interview spaces with witnesses. In Taylor and Dando’s (2018) study a formal rapport-building phase was not included, although an informal and friendly conversation did take place prior to the start of the retrieval interview, during which the interviewer used positive nonverbal behaviours (e.g., eye contact, nodding). These behaviours are argued as being key rapport-building techniques (see Abbe & Brandon, 2014) and studies have found them effective (e.g., Collins et al., 2002; Holmberg & Madsen, 2014; Nahouli et al., 2021; Vallano et al., 2011; see Gabbert et al., 2021, for a review). However, these behaviours were common to all conditions, hence rapport was not manipulated nor was it the focus of the research.

Some research has remotely manipulated rapport, and rapport has been investigated in face-to-face video mediated contexts and during the remote production of facial composite sketches (e.g., Kuivaniemi-Smith et al., 2014; Nash et al., 2014; Nash et al., 2020; Sun, 2014). Although markedly different interview environments, the findings are encouraging, suggesting that rapport can be built remotely, and where this occurs interviewees reveal more sensitive information, and the accuracy of witness accounts improves versus where rapport was absent. Furthermore, interviewees report better concentration and feeling less pressured. In common with traditional in-person face-to-face paradigms, a variety of rapport behaviours were employed including informal and friendly conversation, eye contact, reciprocal conversation, friendly tone, use of first name, and appearing interested and engaged.

Although the wider literature indicates potential benefits of VEs as interviewing spaces and possible advantages of avatar-to-avatar rapport (e.g., Mousas et al., 2018; Saarijärvi & Bratt, 2021; Sutherland, 2020) more research is needed. Here, we report a mock eyewitness study where a cluster of rapport-building behaviours were experimentally manipulated in traditional in-person face-to-face and VE avatar-to-avatar interview contexts. We selected a small number of physical and verbal rapport behaviours described in the applied experimental literature (e.g., Collins et al., 2002; Kieckhaefer et al., 2014; Nahouli et al., 2021; Vallano & Schreiber Compo, 2011), the prevailing professional interviewing guidance (e.g., College of Policing, 2021; Ministry of Justice, 2022), and which were appropriate for use both in a VE which neccesitates the use of a headset, and face-to-face contexts, as follows. In the rapport-present conditions, to begin the process of engagement, the interviewers commenced the rapport phase by offering some non-personal information about themselves, interacting with the participant using open-ended invitations to exchange information about neutral topics. Simultaneously, the interviewers displayed two attentive physical behaviours—namely, looking at interviewees/making eye contact when the interviewee was talking (as appropriate) and nodding when the interviewee spoke and answered questions. Two attentive verbal behaviours were also used—namely, referring to the interviewee by their first name and thanking the interviewee whenever they provided information/answered a question. Both verbal and physical behaviours continued throughout the interview (see Table 1) in the rapport-building conditions only and were absent throughout interviews in the no-rapport conditions. Although data collection was completed prior to the publication of a review of rapport in professional contexts (Gabbert et al., 2021), the rapport behaviours used here are all highlighted as key methods for building rapport.

**Table 1:** Interview phase description

Phase	Overview
1. Explain	Explain the interview/research process prior to the commencement of the interview and offer the opportunity to ask questions.
2. Rapport	Interviewer verbally interacts with the participant using two behaviours: i) Open-ended invitations to exchange information. For example, “*Thank you for coming to the University today. It’s quite tricky to find us; how did you get here?*” ii) Offering some nonpersonal information about themself to begin this process. For example, “*I ride my bike here, but I worry about my bike being stolen. One was damaged last year.*” Interviewer displays two attentive physical behaviours: i) Looking at interviewees/making eye contact when they were talking ii) Nodding when interviewees speak/answer questions Interviewer displays two attentive verbal behaviours: i) Referring to the interviewee by their first name once the interviewee had agreed this would be acceptable ii) Thanking interviewees whenever they provided information and answered a question. For example, “*Thank you, that was useful in helping me to understand*.” In the rapport-present condition, the two physical and two attentive verbal behaviours continued throughout both the free-recall and questioning phases (see Gabbert et al., 2021; Nahouli et al., 2021).
3. No Rapport	The interviewer immediately moves from the Explain phase (Phase 1) to the Free-recall phase. None of the above verbal or physical behaviours are used. This persists throughout the interview.
4. Free-recall	Commenced with an explanation of the four ground rules: 1. Report all/everything 2. Do not guess 3. Say if you do not know 4. Say if you do not understand Participants were then instructed to explain everything they could remember, uninterrupted by the interviewer. The interviewer made bullet-point notes regarding the topics recalled and the order in which they were recalled for use during the questioning phase. Once interviewees had finished, all were asked if they wished to add anything else.
5. Questions	Commenced with a reminder of the four ground rules (above), following which participants were asked one TED prefaced probing question related to each of the topics recalled in the free-recall, one by one. For example, “*You mentioned a girl standing at the bar, please* ***describe*** *that girl to me in as much detail as you can.*”
6. Close	Participants were thanked and offered the opportunity to ask questions.

In light of the extant empirical literature concerning interviewee experience and memory performance, we formulated a number of hypotheses. First, being interviewed in a VE will improve mock witness memory versus an in-person face-to-face interview (H^1^). Further, irrespective of interview context some rapport-building will improve mock witness memory performance (H^2^) since previous research has highlighted the importance of rapport for improved cognition . Finally, irrespective of interview environment rapport-building will have a positive impact on self-reported interview experience (H^3^) because the literature generally reports improved social benefits when rapport is present. We do not hypothesise regarding the impact of rapport as a function of interview environment since relevant literature is sparce and does not support a meaningful hypothesis in this regard. Rather, we investigated rapport across environments by considering interaction effects guided by the following research question—is rapport important and impactful in a virtual environment as the literature suggests it is during face-to-face interactions.

## Method

### Participants

An a priori power analysis using G*Power 3.1 (Faul et al., 2007) indicated that a sample size of 100 mock witnesses would be more than adequate to detect large effects (assuming power = .80 and *a* = .05). Forty-four males and 56 females from the general population participated with a mean age of 25.8 years (*SD* = 7.5), ranging from 18 to 50 years. There were no significant differences in mean age across conditions (rapport & environment), *F* = 1.46, *p* = .23. Participants were recruited through word of mouth, social media, and advertisements placed in the locality of the University.

### Design

A mock witness 2 (environment: face-to-face, virtual) × 2 (rapport: present, absent) design was employed using five interviewers, as typically occurs in real-life cases where there are several witnesses. The mean number of interviews conducted by each interviewer was 20 (ranging from 11 to 32). Participants individually watched a stimulus video and were then randomly allocated to one of the experimental interview conditions. Forty-eight hours later, participants were interviewed according to condition. The dependent variable was memory for the video, measured by the number of correct, incorrect and confabulated information items recalled, and percentage accuracy (correct details as a function of overall details recalled). Immediately post interview, feedback was collected to understand interviewee experience. Ethical approval was obtained from the University of Westminster research ethics review committee.

### Materials

#### Crime stimulus video

A pre-recorded video lasting 1 min 40 seconds of a mock fight in a public bar was viewed individually by participants via a laptop computer (see https://youtu.be/4PumXJX1iZo). The video depicts a man buying drinks for a female friend while another female character walks over to chat about a coursework assignment. The second female character leaves and the male and female then walk to the other side of the bar where they sit down at a table. Their conversation is interrupted by two men, first talking and then shouting. One of the men pushes the other before punching him to the ground and repeatedly punching him. The male friend goes over and states he is unconscious. A woman who is sitting behind them calls an ambulance.

#### Interview protocols

Irrespective of condition, all interviews comprised two retrieval attempts in the same order. First, participants were asked to provide a free-recall account of everything they could remember. This initial account was uninterrupted by the interviewer who made bullet point notes regarding the topics recalled and the order in which they were recalled for use during the questioning phase that followed. In the questioning phase, each of the topics recalled in the preceding free-recall phase were probed in turn using one Tell, Explain, and Describe question per topic. Probing questions commencing with Tell, Explain, and Describe are often referred to as TED questions and are recommended as part of several evidence-based interview protocols. TED questions are open, probing, information gathering questions that prompt the interviewee to elaborate in detail on topics that have been previously mentioned in the initial free-recall prompt (see Kontogianni et al., 2020; Oxburgh et al., 2010). Accordingly, the number of TED questions asked during the questioning phase was predicated on participants’ free-recall (see Dando et al., 2020 ; Fisher & Geiselman, 1992; Vrij et al., 2014).

**Fig. 1:**
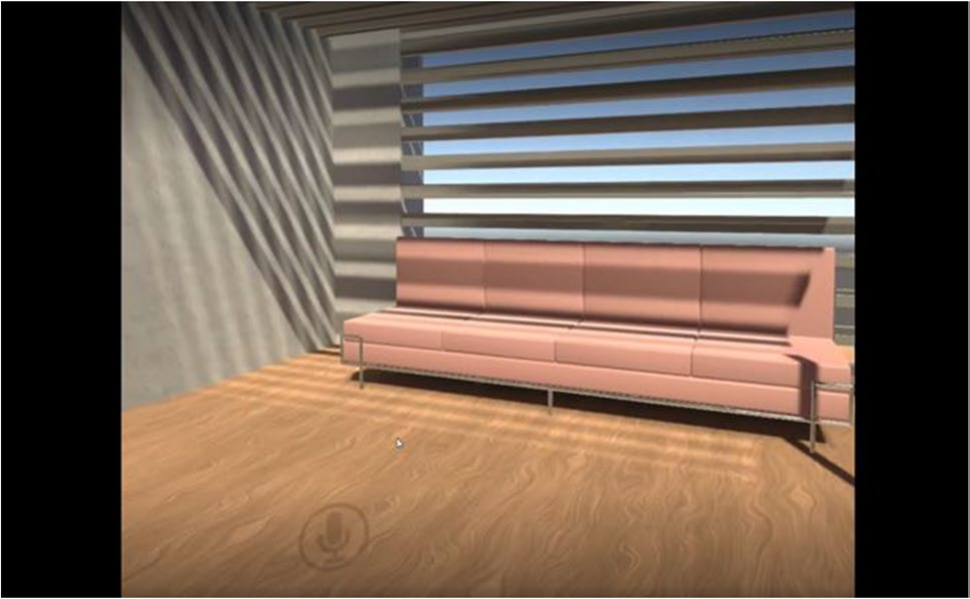
VE environment view at point of entry

The free-recall commenced with a pre-interview explain phase and finished with a closure phase. Participants in the rapport condition experienced an additional rapport phase, with all rapport-building behaviours then continuing throughout the interview. Participants in the no-rapport conditions did not experience the rapport-building phase, and the rapport behaviours were all absent through the entire interview. Interview protocols are outlined in Table 1 (detailed protocols are available from the first author). The questioning phase commenced with a reminder of the four ground rules. Five experienced researchers conducted all the interviews, following the condition appropriate protocols, verbatim (but see Procedure and Fig. 1 also).

#### Post interview questionnaire

All participants completed an anonymous post interview questionnaire within 15 mins of being interviewed. The questionnaire was hosted remotely on Qualtrics. The questionnaire comprised a total of ten questions, however, participants answered questions according to condition (see below). Nine questions used a Likert type scale ranging from 1 to 5 (e.g., 1 = *very easy* to 5 = v*ery hard*; 0% confident to 100% confident etc.), allowing participants to select one of the 5 response options. One question was dichotomous yes/no (full questionnaire available from first author). All participants were asked the following five Likert scale questions: (i) how easy/difficult did you find it to remember the video, (ii) how confident are you that what you remembered was correct, (iii) how confident are you that you did not make any errors, (iv) how comfortable did you feel during the interview, (v) how easy/difficult was it to say when you did not remember, and (vi) how friendly/unfriendly did you feel the interviewer was towards you during the interview. Participants in the VE condition were asked the following three Likert questions relating to the VE and VR headset: (i) how easy/difficult did you find it to use the VR headset, (ii) how comfortable was the VR headset, (iii) overall, how easy or difficult was it to be interviewed in a VE, and (iv) 3 likert scale questions and one final dichotomous yes/no question (have you used a vr headset before- yes/no).

#### Equipment

**Fig. 2:**
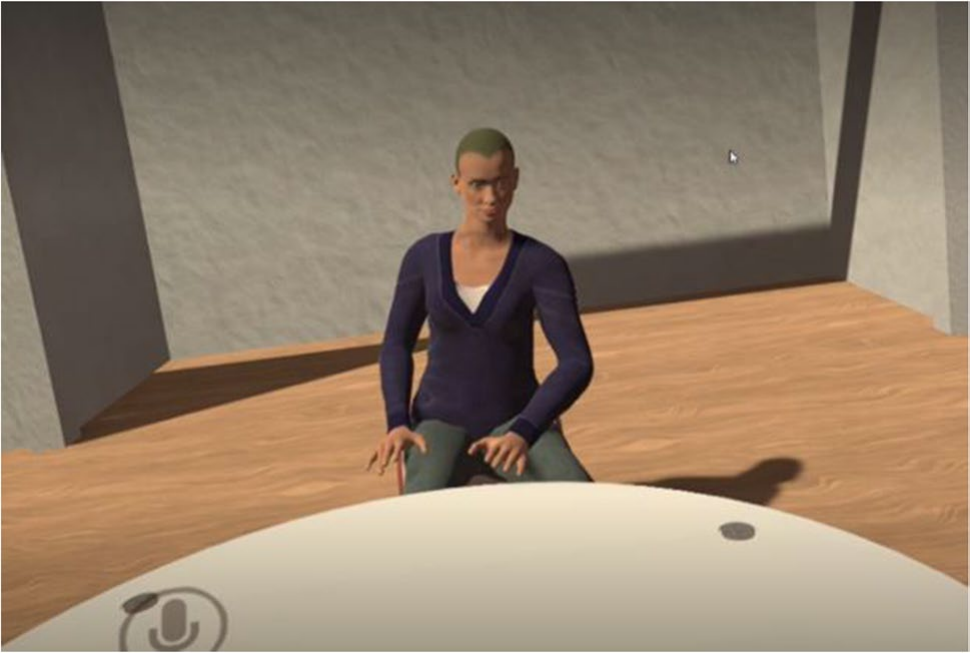
Avatar and environment example

In the VE condition, interviewer and participant were in different rooms within the same building and communicated using an Oculus Rift S virtual reality (VR) headset. The Oculus Rift creates a sense of complete immersion in a three-dimensional world (here, a bespoke interview environment) via 2,560 × 1,440 high-resolution OLED panels, one for each eye, which globally refresh at a rate of 90 Hz. An on-board Inertia Measurement Unit (IMU) positional camera allows transitional and rotational movement to be tracked with 6 DoF. The headset tracks the movements of both head and body, then translates them into VR with realistic precision. Verbal communication was via 3D positional audio built directly into the headset, which was digitally recorded for transcription and coding. A bespoke, virtual interview environment was developed for this research using Unreal Engine 4. The VE interview environment was purposely sparse and neutral, comprising a sofa, a table and chairs—one chair for the avatar interviewer, the other for the avatar participant (see Figs. 1 and 2). Limited choice was offered to participants regarding the appearance of their avatar, likewise the interviewers. They could appear as male or female. Participants and all interviewers chose to match their avatar to their gender appearance.

### Procedure

Participants were recruited to take part in a mock eyewitness research study investigating the use of virtual environments for investigating long term memory performance. The study was advertised via social media, locally around the University, and via word of mouth. Interested participants were able to contact the researchers, at which point they were provided with an information sheet and consent form, which outlined some inclusion criteria, including being over 18 years of age, and not ever having been interviewed as a witness or victim of crime. Once participants had met the inclusion criteria and then consented to participate, they accessed a one-time-only link which allowed them to view the stimulus video. They were interviewed about the video 48 hours later. In the UK and elsewhere, other than for the most serious crimes, witnesses are not usually interviewed immediately (see Hoogesteyn et al., 2020; Hope et al., 2011). Rather, for more common “volume” crime events such as depicted in the stimulus video used here, delays in interviewing can often range from several hours to several days. Hence, as is common practice in research of this nature, we too introduced a delay to enhance the ecological validity. Prior to interview, participants were randomly assigned to one of the interview conditions (face-to-face rapport, face-to-face no rapport, VE rapport, VE no rapport) and interviewed accordingly. Participants completed the post interview feedback questionnaire within 15 mins. Participants took part voluntarily and received no payment or other compensation for their time.

Interviewers were all experienced researchers in the domain of experimental investigative interviewing. Since rapport is not a singular concept, but rather comprises a cluster of behaviours which are variously understood and applied according to context, prior experience, and training, before conducting interviews for this research all underwent bespoke (designed for this research by the first author) training towards reducing variability of application. Training adopted a collaborative pedagogical approach and comprised (i) a 4-hour long classroom-based introduction to the rapport behaviours that were the subject of this research, including how and when they should be used during interviews; (ii) 2 × 4-hour long instruction and practice sessions using the VE and VR headsets; (iii) reading of theoretical and applied training materials produced for this research; (iii) practice interviews (eight in total, four in each environment) face-to-face and using the VR, which were digitally recorded to allow feedback and evaluation on each interview before moving to the next; and (iv) instruction on reflective research practice and critical self-evaluation of performance. Once researchers had attended the training sessions and completed the required competencies (consistent and correct application of the rapport behaviours as required by the protocols in at least two of the four practice interviews per environment), they were able to commence research interviews. In total, training for this research took between 3 and 4 days to complete.

### Interview coding

Interviews were digitally audio and video recorded, transcribed verbatim, and coded for correct, erroneous (information relevant to the witnessed episode but described with error, e.g., describing a person’s brown jacket, but stating that it was black instead of brown), or confabulated (reporting information that was not present in the film) information recalled. The position in the interview the information was recalled was coded (i.e., whether recalled in the free-recall or questioning phase). Items recalled were only scored once (i.e., repetitions were not scored irrespective of interview phase). Five interviews from each condition (20 in total) were randomly selected for recoding by an independent coder blind to the aims and hypotheses of the research but familiar with the method of scoring. Two-way mixed effects intraclass correlation coefficient (ICC) analysis testing for absolute agreement between coders for the overall amount of correct, erroneous, and confabulated recall were conducted. Mean estimations with 95% CI reveal very good interrater reliability for correct information, ICC = .993 (95% CI [0.982, 0.994]), errors, ICC = .954 (95% CI [0.888, 0.982]) and confabulations, ICC = .865 (95% CI [0.658, 0.946]).

The same sample of 20 interviews were coded by a further two independent coders blind to the aims and hypotheses of the research for adherence to the interview protocol as a function of condition: that is, no rapport-building behaviours in the rapport-absent (control) conditions and presence of rapport-building behaviours in the rapport-present conditions (see Table 1). A scoring sheet was used where each of the behaviours were coded, ranging from 1 to 3 for each according to condition (e.g., 3 = *fully implemented the open-ended self-disclosure behaviour*, 2 = *partially implemented the open-ended questions behaviour*, 1 = *did not implement*) as a function of phase (e.g., see Nahouli et al., 2021). The rapport phase occurred only in the rapport-building condition, while the free-recall and questioning phases were common to all conditions. In the rapport phase, six rapport behaviours were coded (see Table 1), and in the free-recall and questioning phases, four rapport behaviours were coded. To score 1, the behaviour in question had to be absent. To score 2, the behaviour had to be present at least once but no more than twice. To score 3, the behaviour had to be present at least three times. Thus, each phase was awarded scores ranging from 6 to 18 for the rapport phase (in the rapport condition, only), and ranging from 4 to 12 for each of the free-recall and questioning phases.

Two-way mixed-effects ICC analysis testing for absolute agreement between coders for the six rapport-building behaviours expected to be present/absent in the rapport phase revealed good interrater reliability for each of the behaviours; open questions, ICC = .899 (95% CI [.593, .975]), offering non-personal information, ICC = .862 (95% CI [.443, .966]), making eye contact, ICC = .862 (95% CI [.443, .966]), nodding, ICC = .865 (95% CI [.498, .964]), referring to interviewee by name, ICC = 1.00 (95% CI [1.00, 1.00]) and thanking the interviewee, ICC = .757 (95% CI [.096, .935]).

Good interrater reliability was also found for the four rapport-building behaviours expected to be present/absent in the free-recall phase: eye contact, ICC = .938 (95% CI [.843, .975]), nodding, ICC = .883 (95% CI [.705, .954]), referring to interviewee by name, ICC = 1.00 (95% CI [1.00, 1.00]), and thanking the interviewee, ICC = 1.00 (95% CI [1.00, 1.00]); and questioning phase: making eye contact, ICC = .883 (95% CI [.705, .954]), nodding, ICC = .979 (95% CI [.948, .992]), referring to interviewee by name, ICC = 1.00 (95% CI [1.00, 1.00]), and thanking the interviewee, ICC = 1.00 (95% CI [1.00, 1.00]).

### Rapport manipulation analysis

Means (*SD*s & 95% CIs) for rapport behaviours across phases common to all interview conditions as a function of environment and interview condition are displayed in Table 2.

**Table 2:** Mean scores for presence/absence of rapport as a function of condition and environment, as a function of recall phase (1 = *not implemented*; 2 = *partially implemented*; 3 = *fully implemented*)

	Mean (SD) 95% CI	
	Free Recall	Questioning
VE Rapport Absent		
Looking/eye contact	1.16 (.37) 1.01; 1.32	1.16 (.36) 1.00; 1.31
Nodding	1.08 (.27) .97; 1.19	1.08 (.27) .97; 1.19
Name	1.00 (.00) 1.00; 1.00	1.04 (.20) .96; 1.12
Thank you	1.08 (.27) .97; 1.19	1.12 (.33) .98; 1.25
F-to-F Rapport Absent		
Looking/eye contact	1.16 (.38) 1.01; 1.31	1.17 (.37) 1.01; 1.31
Nodding	1.08 (.30) .96; 1.19	1.08 (.27) .97; 1.19
Name	1.04 (.20) .96; 1.12	1.04 (.20) .96; 1.12
Thank you	1.09 (.29) .97; 1.19	1.08 (.27) .96; 1.19
VE Rapport Present		
Looking/eye contact	2.80 (.50) 2.59; 3.01	2.72 (.54) 2.50; 2.94
Nodding	2.84 (.37) 2.65; 2.99	2.88 (.33) 2.74; 3.02
Name	2.84 (.47) 2.64; 3.04	2.80 (.51) 2.59; 3.01
Thank you	2.80 (.50) 2.59; 3.00	2.80 (.50) 2.59; 3.01
F-to-F Rapport Present		
Looking/eye contact	2.88 (.33) 2.74; 3.01	2.89 (.34) 2.74; 3.02
Nodding	2.88 (.34) 2.74; 2.99	2.88 (.33) 2.92; 3.03
Name	3.00 (.00) 3.00; 3.00	2.96 (.20) 2.88; 3.04
Thank you	3.00 (.00) 3.00; 3.00	2.96 (.21) 2.87; 3.04

The rapport-present main effect was nonsignificant for all four rapport-building behaviours across environments, in both the free-recall, all *F*s < 4.00, all *p*s > .059, and questioning phases, all *F*s < 2.21, all *p*s > .144 revealing that all behaviours were similarly present across the two environments in both recall phases. Likewise, there were no significant differences across environments in the rapport-absent conditions for the four rapport-building behaviours in either the free-recall, all *F*s < 1.00, all *p*s > .322, or questioning phases, all *F*s < .214, all *p*s > .646, and so all rapport behaviours were similarly absent.

There were no significant differences across environment (VE and face-to-face) in the rapport phase of the rapport-present conditions for use of the six rapport behaviours applied in this phase: (i) open-ended questions (*M*_VE_ = 2.80, *SD* = .41; *M*_FtF_ = 2.84, *SD* = .37), (ii) offering information (*M*_VE_ = 2.96, *SD* = .20; *M*_FtF_ = 2.91, *SD* = .21), (iii) looking at interviewees when they speak (*M*_VE_ = 2.80, SD = .40; *M*_FtF_ = 2.92, *SD* = .28), (iv) using interviewee’s name (*M*_VE_ = 2.88, *SD* = .33; *M*_FtF_ = 2.88, *SD* = .32), (v) nodding in acknowledgement when interviewees speak (*M*_VE_ = 2.84, *SD* = .37; *M*_FtF_ = 2.95, *SD* = .20), and (vi) thanking interviewees when they answer questions (*M*_VE_ = 2.84, *SD* = .31; *M*_FtF_ = 2.88, *SD* = .31), all *F*s < 2.00, all *p*s > .164.

There was a significant main effect of rapport condition (present/absent) for all four rapport behaviours in the free-recall phase (common to all interviews), looking at the interviewee/making eye contact when the interviewee speaks, *F*(1, 96) = 3221.94, *p* < .001, η_p_^2^ = .91, using the interviewee’s name, *F*(1, 96) = 5010.78, *p* < .001, η_p_^2^ = .94, thanking the interviewee when they answered questions, *F*(1, 96) = 1319.51, *p* < .001, η_p_^2^ = .993, and nodding in acknowledgement when the interviewee spoke, *F*(1, 96) = 1514.70, *p* < .001, η_p_^2^ = .94. All behaviours occurred significantly more often in the rapport-present condition than in the rapport-absent condition. There were no significant main effects of environment, and the Environment × Rapport interactions were also nonsignificant, all *F*s < 3.08, all *p*s > .082 (see Table 3). Similarly, in the questioning phase (common to all interviews) there was a significant main effect for all four rapport behaviours (Table 3), looking at the interviewee/making eye contact when they spoke, *F*(1, 96) = 1514.07 *p* < .001, η_p_^2^ =.94, using the interviewee’s name, *F*(1, 96) = 2353.02, *p* < .001, η_p_^2^ = .96, thanking the interviewee, *F*(1, 96) = 3225.94, *p* < .001, η_p_^2^ = .97, and nodding in acknowledgement, *F*(1, 96) = 1412.10 , *p* < .001, η_p_^2^ = .92.

**Table 3:** Mean scores for presence/absence of rapport-building behaviours as a function of environment and rapport across phases (1 = *not implemented*; 2 = *partially implemented*; 3 = *fully implemented*)

	Mean (SD) 95% CI	
	Free Recall	Questioning
Virtual Environment (VE)		
Looking/eye contact	1.98 (.98) 1.93. 2.03	2.00 (.98) 1.93. 2.07
Nodding	2.00 (1.10) 1.93, 2.07	1.98 (.98) 1.91, 2.05
Name	1.96 (.99) 1.92, 1.99	1.99 (1.00) 1.92, 2.03
Thank you	2.00 (.99) 1.93, 2.06	1.98 (.99) 1.93, 2.02
Face-to-Face (FtF)		
Looking/eye contact	2.00 (1.01) 1.93, 2.05	2.00 (1.01) 1.93, 2.06
Nodding	2.00 (.99) 1.03, 2.06	2.02 (.96) 1.95, 2.09
Name	2.00 (1.03) 1.92, 1.99	1.98 (.97) 1.92, 2.36
Thank you	1.99 (.97) 1.93, 2.07	1.96 (.98) 1.91, 2.01
Rapport Absent		
Looking/eye contact	1.02 (.14) .97, 1.07	1.06 (.24) .99, 1.13
Nodding	1.06 (.19) .99, 1.13	1.06 (.23) .99, 1.12
Name	1.00 ( 0.00) .96, 1.04	1.02 ( .14) .96, 1.08
Thank you	1.07 (.24 ) .99, 1.12	1.00 (0.00) .95, 1.05
Rapport Present		
Looking/eye contact	2.96 (.20) 2.91, 3.01	2.94 (.24) 2.87, 3.01
Nodding	2.94 (.24 ) 2.87, 3.01	2.94 (.23 ) 2.87, 3.01
Name	2.96 (.20 ) 2.92, 3.00	2.94 (.20 ) 2.89, 2.98
Thank you	2.94 (.24) .96, 1.04	2.94 (.24) 2.88, 3.00

All behaviours occurred significantly more often in the rapport condition than in the no-rapport condition. There were no significant main effects of environment, and the Environment × Rapport interactions were also nonsignificant, all *F*s < 1.02, all *p*s > .315.

A random sample of five interviews conducted by each interviewer (25 interviews in total) was coded for adherence to the interview protocol phases by two independent raters using a scoring sheet (ranging from 3 = *fully implemented every phase* to 0 = *did not implement*). The rapport-present interviews comprised six phases (see Table 1), whereas the no-rapport interviews comprised five phases. Analysis revealed a substantial level of agreement between raters, Kappa = .921, *p* = .003. Interviewer adherence across phases revealed no significant differences as a function of interviewer for adherence to each phase, all *F*s < 3.211, *p* = > .217, and each interviewer applied each phase as a function of condition.

## Results

### Analysis approach

To investigate H^1^ and H^2^ a series of 2 (environment: face-to-face, virtual) × 2 (rapport: present, absent) ANOVAs were conducted. Global memory performance (performance across the duration of the interview) main effects and interactions were investigated using the number of correct, incorrect, and confabulated items recalled, and percentage accuracy. Performance as a function of retrieval phase was analysed to investigate the pattern of memory performance and locus of any significant global main effects and interactions. Finally, to investigate H^3^, interviewee post interview feedback was analysed across conditions where appropriate, and responses to condition-specific questions. Guided by our additional research question, Environment × Rapport interactions were exploratory, allowing us to investigate the combined effects of rapport and retrieval environment. Global duration of interviews, duration of the two recall phases (combined), and the number of questions asked in the question phase of interviews were also analysed to fully explore the impact of environment and condition for applied audiences.

### Global memory performance

#### Correct recall

There were significant main effects of environment, *F*(1, 96) = 17.814, *p* < .001, η_p_^2^ = .16, and rapport, *F*(1, 96) = 6.840, *p* = .010, η_p_^2^ = .07, for correct recall. Participants interviewed in the VE recalled more correct information than those interviewed face-to-face, and when rapport was present participants recalled more correct information (see Table 4 for main effects). There was a significant Environment × Rapport interaction, *F*(1, 96) = 6.638, *p* = .012, η_p_^2^ = .07. When rapport was present, participants interviewed in the VE recalled more correct information, *p* < .001 (Table 5). All other interactions were nonsignificant.

**Table 4:** Main effects of rapport and environment (Means, *SD*s, and 95% CIs) on correct, incorrect and confabulated recall

	Correct	Incorrect	Confabs
	M (SD) 95% CI		
Environment			
Virtual Environment	72.94 (19.88)	6.44 (4.54)	.68 (0.95)
	68.33, 77.55	4.95, 7.93	0.23, 1.13
In-Person FtF	62.92 (14.02)	10.20 (6.76)	1.20 (2.13)
	58.31, 67.53	8.71, 11.69	.79, 1.69
Rapport			
Present	71.02 (18.16)	7.00 (4.11)	.64 (.95)
	66.68, 75.36	5.51, 8.49	.19, 1.09
Absent	62.94 (16.04)	9.64 (7.28)	1.24 (2.14)
	58.60, 67.28	8.09, 11.13	.79, 1.69

**Table 5:** Rapport × Environment interactions (Means, *SD*s, and 95% CIs) for global correct, incorrect, and confabulations

	Correct	Incorrect	Confabs
M (SD) 95% CI			
Virtual Environment + Rapport	81.52 (19.48) 75.39, 87.65	7.04 (3.67) 4.93, 9.15	.76 (.91) .13, 1.40
FtF + Rapport	60.52 (7.93) 54.39, 66.65	6.96 (4.61) 4.85, 9.07	.52 (0.71) -.13, 1.15
Virtual Environment No Rapport	65.48 (17.16) 59.35, 71.61	5.84 (5.31) 3.73, 7.95	.60 (0.91) -.04, 1.24
FtF No Rapport	60.48 (14.75) 59.35, 71.61	13.44 (7.05) 11.33, 15.54	1.88 (2.80) 1.25, 2.52

#### Incorrect recall

There were significant main effects of environment, *F*(1, 96) = 12.541, *p* < .001, η_p_^2^ = .12, and rapport, *F*(1, 96) = 6.183, *p* = .015, η_p_^2^ = .06, for incorrect recall. Participants interviewed in the VE recalled fewer incorrect items of information than those interviewed in-person face-to-face (see Table 4). When rapport was present participants also recalled fewer incorrect items of information. There was a significant Environment × Rapport interaction, *F*(1, 96) = 13.081, *p* < .001, η_p_^2^ = .12 (see Table 5). When rapport was absent, participants interviewed FtF recalled significantly more incorrect items of information, *p* < .001. All other interactions were nonsignificant.

#### Confabulations

There were nonsignificant main effects of environment and rapport, all *F*s < 3.52 and all *p*s > .064. There was a significant Environment × Rapport interaction, *F*(1, 96) = 5.648, *p* = .019, η_p_^2^ = .56. When rapport was absent, participants confabulated more during in-person face-to-face interviews, *p* =.002 (see Table 5). All other interactions were nonsignificant.

#### Percentage accuracy

There were significant main effects of environment, *F*(1, 96) = 21.069, *p* < .001, η_p_^2^ = .18, and rapport, *F*(1, 96) = 11.350, *p* = .001, η_p_^2^ = .11, for percentage accuracy. Participants were more accurate in the VE (*M*_*VE*_ = 91.23, *SD* = 3.11, 95% CI [88.79, 93.66]) than in-person face-to-face (*M*_*F2F*_ = 89.69, *SD* = 6.26, 95% CI [87.26, 93.67]). They were also more accurate when rapport was present (*M*_*rapport*_ = 90.46, *SD* = 4.95, 95% CI [89.06, 91.87]) than when it was absent (*M*_*no rapport*_ = 86.33, *SD* = 8.58, 95% CI [83.89, 88.77]). The Environment × Rapport interaction was also significant, *F*(1, 96) = 11.118, *p* = .001, η_p_^2^ = .10. Participants were less accurate when interviewed in-person face-to-face when rapport was absent (*M*_*F2F No rapport*_ = 81.48, *SD* = 7.99, 95% CI [79.05, 83.91]) than those interviewed in-person face-to-face when rapport was present (*M*_*F2F rapport*_ = 89.69, *SD* = 3.11, 95% CI [87.26, 92.13]) and those interviewed in the VE when rapport was present (*M*_*VE rapport*_ = 91.23, *SD* = 6.12, 95% CI [88.80, 93.67]) and absent (*M*_*VE No rapport*_ = 91.19, *SD* = 6.12, 95% CI [88.76, 93.63]), all *p*s < .001. All other interactions were nonsignificant.

### Recall phase memory performance

Given the small mean confabulations, in analysing memory as a function of interview phase (free-recall and questioning) we collapsed the two types of errors (incorrect information and confabulations) to allow a more meaningful interpretation.

### Free-recall phase

#### Correct recall

There was a significant main effect of environment, *F*(1, 96) = 13.81, *p* < .001, η_p_^2^ = .13. Participants interviewed in the VE recalled more correct items of information in the free-recall phase (*M*_*VE*_ = 41.96, *SD* = 13.32, 95% CI [39.04, 44.88]) than those interviewed in-person face-to-face (*M*_*F2F*_ = 34.22, *SD* = 7.14, 95% CI [26.79, 38.05]). The main effect of rapport was nonsignificant, *F* = 3.01, *p* = .082 (*M*_*rapport*_ = 39.92, *SD* = 11.97; *M*_*no rapport*_ = 36.26, *SD* = 10.45). The Environment × Rapport interaction was significant, *F*(1, 96) = 4.18, *p* = .044, η_p_^2^ = .04. When rapport was present participants interviewed in the VE recalled more correct information in the free-recall phase (*M*_*VE rapport*_ = 45.92, *SD* = 12.83, 95% CI [41.79, 50.05]) than those interviewed face-to-face (*M*_*F2F rapport*_ = 33.92, *SD* = 7.26, 95% CI [29.79, 38.05]), *p* < .001. When rapport was absent, participants interviewed in the VE recalled more correct information (*M*_*VE No rapport*_ = 38.00, *SD* = 12.85, 95% CI [33.87, 42.13]) than those interviewed face-to-face (*M*_*F2F No rapport*_ = 34.52, *SD* = 7.16, 95% CI [30.87, 38.65]), *p* = .001.

#### Errors

There were significant main effects of environment, *F*(1, 96) = 18.23, *p* < .001, η_p_^2^ = .16, and rapport, *F*(1, 96) = 8.63, *p* = .004, η_p_^2^ = .08 for the number of errors in the free-recall. Participants interviewed in the VE made significantly fewer errors (*M*_*VE*_ = 1.92, *SD* = 2.44, 95% CI [1.20, 2.64]) than those in the in-person face-to-face condition (*M*_*F2F*_ = 4.10, *SD* = 3.18, 95% CI [3.83, 4.18]). Where rapport was present participants made significantly fewer errors (*M*_*rapport*_ = 2.26, *SD* = 2.04, 95% CI [1.54, 2.98]) than when rapport was absent (*M*_*No rapport*_ = 3.76, *SD* = 3.62, 95% CI [3.04, 4.48]). The Environment × Rapport interaction was significant, *F*(1, 96) = 16.27, *p* < .001, η_p_^2^ = .15. When rapport was absent, participants interviewed in-person face-to-face made more errors (*M*_*F2F No rapport*_ = 5.88, *SD* = 3.14, 95% CI [4.87, 6.89]) than those interviewed in the VE (*M*_*VE No rapport*_ = 1.64, *SD* = 1.60, 95% CI [.67, 2.65]), *p* = .001, and when rapport was present participants interviewed in-person face-to-face (*M*_*F2F rapport*_ = 2.32, *SD* = 2.06, 95% CI [1.31, 3.33]) made more errors than this interviewed in the VE (*M*_*VE rapport*_ = 1.64, *SD* = 1.08, 95% CI [1.89, 3.21]), *p* < .001.

### Questioning phase

#### Correct recall

There were significant main effects of environment and rapport on the amount of correct information recalled in the questioning phase, *F*(1, 96) = 7.51, *p* = .007, η_p_^2^ = .07, and *F*(1, 96) = 4.42, *p* = .038, η_p_^2^ = .04, respectively. Participants interviewed in the VE recalled more correct information (*M*_*VE*_ = 31.54, *SD* = 11.73, 95% CI [28.72, 34.36]) than those in the in-person face-to-face condition (*M*_*F2F*_ = 26.04, *SD* = 8.76, 95% CI [23.22, 28.86]). Participants also recalled more correct information when rapport was present (*M*_*rapport*_ = 30.09, *SD* = 12.30, 95% CI [28.08, 33.72]) than when it was absent (*M*_*No rapport*_ = 26.68, *SD* = 8.32, 95% CI [23.86, 29.50]). The Environment × Rapport interaction was nonsignificant for the questioning phase, *F* = 3.78, *p* = .055.

#### Errors

The main effects of environment, *F* = 3.86, *p* = .052 (*M*_*F2F*_ = 7.10 *SD* = 5.98, 95% CI [5.76, 8.44]; *M*_*VE*_ = 5.22 *SD* = 3.60, 95% CI [5.76, 8.44]), and rapport, *F* = 2.52, *p* = .116, were nonsignificant for the number of errors in the questioning phase (*M*_*Rapport*_ = 5.40 *SD* = 3.49 95% CI [5.76, 8.44]; *M*_*No rapport*_ = 6.92 *SD* = 6.09, 95% CI [5.76, 8.44]). However, the Environment × Rapport interaction was significant, *F*(1, 96) = 5.67, *p* = .019, η_p_^2^ = .06. Participants reported more errors in the in-person face-to-face condition when rapport was absent (*M*_*F2F no rapport*_ = 9.00 *SD* = 7.11, 95% CI [7.10, 10.90]) than in the VE when rapport was absent (*M*_*VE no rapport*_ = 4.84 *SD* = 4.03, 95% CI [2.94, 6.74]), *p* = .004. When rapport was present, there were no significant differences between participants in the in-person face-to-face (*M*_*F2F rapport*_ = 5.20 *SD* = 3.85, 95% CI [2.94, 6.74]) and VE conditions (*M*_*VE rapport*_ = 5.60 *SD* = 3.16, 95% CI [3.70, 7.50]).

### Interview duration

#### Global duration

As one might expect due to the additional rapport phase, there was a significant main effect of condition on interview duration (from start to finish including all interview phases), *F* (1, 96) = 108.22, *p* = <. 001, η^2^ = .53. Globally, rapport-building interviews were significantly longer (*M* = 44.96 mins, *SD* = 5.12, 95% CI [42.60, 46.17]) than the no-rapport-building interviews (*M* = 31.17 mins, *SD* = 9.15, 95% CI [29.38, 32.95]). The main effect of environment was nonsignificant, *F* (1, 96) = 1.13, *p* = . 291, as was the Environment × Condition interaction, *F*(1, 96) = .02, *p* = . 887.

#### Recall phase duration

Similarly, the duration of the free-recall and question phases combined, revealed a significant main effect of condition, *F*(1, 96) = 73.17, *p* <. 001, η^2^ = .43. As expected, rapport-building interviews were longer across the combined recall phase (*M* = 32.71 mins, *SD* = 5.56, 95% CI [31.33, 34.10]) than the no rapport interviews (*M* = 24.29 mins, *SD* = 4.09, 95% CI [22.91, 25.67]). The main effect of environment was nonsignificant, *F*(1, 96) = .35, *p* = . 553, as was the Environment × Condition interaction, *F*(1, 96) = .09, *p* = . 759.

### Probing tell, explain, describe (TED) questions

The main effect of rapport (*M*_*rapport*_ = 11.52, *SD* = 1.76, *M*_*no rapport*_ = 10.74, *SD* = 2.16), was nonsignificant, *F* = 3.822, *p* = .053, as was the main effect of environment (*M*_*VE*_ = 11.16, *SD* = 2.08, 95% CI [10.60, 11.72]; *M*_*F2F*_ = 11.10, *SD* = 1.94, 95% CI [10.54, 11.66]), *F* = .03, *p* = .881. The Environment × Rapport interaction for the number of questions asked was also nonsignificant, *F* = .003, *p* = .961.

### Post interview feedback

Feedback means (*SD*s and 95% Cis) are shown in Table 6. There was a significant main effect of rapport, *F*(1, 96) = 16.86, *p* < .001, η_p_^2^ = .15, and a significant Rapport × Environment interaction, *F*(1, 96) = 5.590, *p* = .020, η_p_^2^ = .06, for how easy/difficult participants had found it to remember what they had seen. Participants in the rapport-present condition reported finding it easier to remember than those in the no-rapport condition. Participants in the no rapport VE condition reported finding the recall task more difficult than those in the no rapport face-to-face condition. The effect of environment was nonsignificant, *F* = 2.17, *p* = .109.

**Table 6:** Post interview feedback main effects of environment and rapport, and interaction means (*SD*s & 95% CI)

	Mean (SD) 95% CI							
	Rapport	No Rapport	VE	F-to-F	VE + Rapport	VE No Rapport	F-to-F. + Rapport	F-to-F No Rapport
Easy/Difficult to remember (1 = very easy; 5 = very hard )	2.26 (.75) 2.03, 2.49	2.92 (.90)**. 2.69, 3.15	2.72 (.97) 2.49, 2.95	2.46 (.79) 2.23, 2.69	2.20 (.71) 1.88, 2.52	3.24 (.93)** 2.91, 3.56	2.32 (.80) 2.00, 2.64	2.60 (.76) 2.81, 2.92
Confident correct (1 = not at all; 5 = extremely)	3.58 (.81)**. 3.37, 3.79	2.66 (.92) 2.52, 2.87	3.54 (.81)**. 3.33, 3.75	2.66 (.94) 2.52, 2.76	3.88 (.78) 3.59, 4.17	3.20 (.76) 2.90, 3.94	3.28 (.74) 2.99, 3.57	2.04 (.68) 1.75, 2.33
Confident made no errors (1 = extremely; 5 not at all)	3.10 (.91) 2.84, 3.36	2.94 (.94) 3.68, 3.20	2.94 (.93) 2.68, 3.12	3.10 (.90) 2.84, 3.40	3.12 (.93) 2.75, 3.49	2.76 (.93) 2.39. 3.13	3.08 (.91) 2.71, 3.45	3.12 (.93) 2.75, 3.49
Comfortable (1 = extremely; 5 not at all)	2.18 (1.17)**. 1.97, 2.39	2.52 (1.21). 2.31, 2.73	1.42 (.58)**. 1.21, 1.63	3.28 (.90) 3.07, 3.49	1.28 (.54) .98, 1.58	1.56 (.58) 1.26, 1.86	3.08 (.91) 2.78, 3.38	3.48 (.87) 3.18, 3.78
Say I can't remember (1= difficult; 5 easy)	3.04 (1.51). 2.76, 3.32	3.52 (1.10)* 3.24, 3.80	4.04 (1.02)**. 3.76, 4.32	2.52 (1.09). 2.24, 2. 80	4.08 (1.22). 3.68, 4.48	4.00 (.82) 3.60, 4.40	2.00 (.96)*. 1.60, 2.40	3.04 (.98)* 2.64, 3.44
Friendly (1 = very; 5 = not at all)	2.00 (.95)**. 1.72, 2.28	2.90 (1.27). 2.63, 3.18	2.04 (1.01)**. 1.77, 2.32	2.90 (1.25). 2.63, 3.18	1.92 (.99) 1.53, 2.51	2.16 (1.02)** 1.77, 2.55	2.08 (.91) 1.69, 2.47	3.72 (.98) 3.33, 4.11

* *p* < .05

** *p* < .001

There were significant main effects of rapport, *F*(1, 96) = 42.09, *p* = < .001, η_p_^2^ = .30, and environment, *F*(1, 96) = 35.31, *p* < .001, η_p_^2^ = .27, for confidence that recall was correct. Participants in the rapport-present condition reported feeling more confident their recall was correct than those in the no-rapport condition. Likewise, participants in the VE condition reported feeling more confident they were correct. The Environment × Rapport interaction was also nonsignificant, *F* = 3.57, *p* = .062.

There were no significant main effects nor interactions for confidence ratings regarding errors, all *F*s < 1.17, all *p* > .281. Participants were generally undecided (50%) as to whether they had made any errors or not. There were significant main effects of environment, *F*(1, 96) = 155.83, *p* < .001, η_p_^2^ = .62, and rapport, *F*(1, 96) = 5.21, *p* = .025, η_p_^2^ = .05, for how comfortable participants felt during the interviews. Participants interviewed in the VE reported feeling more comfortable throughout the interview than those interviewed face-to-face, and participants in the rapport condition reported feeling more comfortable than those in the no-rapport condition, *F*(1, 96) = 155.83, *p* < .001, η_p_^2^ = .62. The Environment × Rapport interaction was nonsignificant, *F* = .162, *p* = .688.

There were significant main effects of environment, *F*(1, 96) = 57.28, *p* < .001, η_p_^2^ = .37, and rapport, *F*(1, 96) = 5.71, *p* = .019, η_p_^2^ = .07, for how easy/difficult it was for participants to tell the interviewer they could not remember. Participants in the VE found it easier than those in the face-to-face environment, and participants in the no-rapport condition found it more difficult to say they could not remember. There was a significant Environment × Rapport interaction, *F*(1, 96) = 7.78, *p* = .006, η_p_^2^ = .08. Participants in the face-to-face + rapport condition found it more difficult to say they could not remember than those in the face-to-face no-rapport condition, and participants in both VE + rapport and VE no-rapport conditions found it significantly less difficult than those in the face-to-face + rapport and face-to-face no-rapport conditions, *p =* .002

There were significant main effects of environment, *F*(1, 96) = 19.27, *p* < .001, η_p_^2^ = .18, and rapport, *F*(1, 96) = 23.03, *p* < .001, η_p_^2^ = .19, for ratings of how friendly participants found the interviewer. Participants in the VE found the interviewer more friendly than those in the face-to-face condition, and participants in the rapport condition found the interviewer more friendly than those in the no-rapport condition. A significant Environment × Rapport interaction also emerged, *F*(1, 96) = 12.77, *p* < .001, η_p_^2^ = .12. Participants in the VE no rapport found the interviewer more friendly than those in the no rapport face-to-face rapport condition, *p* = .001.

Participants interviewed in the VE were asked four additional questions. Eight (16%) stated they had used a virtual reality (VR) headset before (yes/no) and 42 (84%) stated they had not. Participants were asked how easy/difficult they found it to use the headset. Overall participants reported finding the headset extremely/somewhat easy to use (*M* = 1.72, *SD* = .83) with no significant difference between the rapport-present (*M* = 1.84, *SD* = .99) and rapport-absent conditions (*M* = 1.60, *SD* = .67), *F* = 1.36, *p* = .314. When asked how they generally liked/disliked being interviewed in a VE, overall participants reported somewhat liking the environment (*M* = 2.02, *SD* = .96) with no significant difference between the rapport-present (*M* = 1.96, *SD* = .90) and rapport-absent conditions (*M* = 2.08, SD = 1.04), *F* = .19, *p* = .663. Simple linear regression revealed that having used a VR headset before was not a significant predictor of ratings of ease of use nor liking of the VE environment, *R*^2^ = .047, *F* = 1.15, *p* = .372. Finally, participants reported the headset to be very comfortable to wear (*M* = 1.62, *SD* = .71).

## Discussion

Theoretical and evidence-based witness interviewing techniques typically champion rapport-building to reduce some of the social demands of recalling a crime event, thereby potentially increasing cognitive capacity for remembering. Cognitive and social benefits have also emerged in remote interview contexts with reduced anxiety and social pressure contributing to improved eyewitness performance. To date, as far as we are aware no research has investigated the combined impact of interview context and rapport-building behaviours. Here, we investigated episodic memory in mock-eyewitness interviews conducted in virtual environments (VE) and in-person face-to-face (FtF), where rapport-building behaviours were either present or absent.

To summarise, participants interviewed in the VE demonstrated superior memory performance to those interviewed FtF, recalling an average of 15% more correct information, reporting more than 50% fewer erroneous details and over 40% fewer confabulations. Further, irrespective of environment, participants recalled more episodic information when rapport was present. However, the VE superiority effect was apparently augmented by the presence of rapport-building behaviours, since participants in the VE + rapport outperformed participants in all other conditions suggesting the benefits of each manipulation were complimentary. As one would expect both the global duration and combined recall phase duration of rapport-present interviews were significantly longer than when rapport was absent. Analysis of the combined recall phase during which participants were retrieving and verbalising episodic information revealed they were 30% longer in rapport-present interviews. However, the number of probing questions asked in the question phase that immediately follows the initial free recall did not significantly differ as a function of rapport, potentially indicating cognitive benefits in terms of supporting cognitive effort whereby responses to questions were more detailed and that the information was accurate since there was no increase in errors and confabulations.

Participants interviewed in the VE reported more correct information, fewer errors and with greater accuracy. Accordingly, our findings support H^1^ and are similar to the results of research conducted by others (e.g., Bethel et al., 2013; Fängström et al., 2017; Hamilton et al., 2017; Nash et al., 2014; Taylor & Dando, 2018). However, our findings add to the emerging literature on cognition in VEs where information gathering is a primary goal (e.g., Baccon et al., 2019; Hope et al., 2011; Nash et al., 2014; Nash et al., 2020; Sun, 2014). The importance of social context is increasingly recognized in applied cognition research (Fisher et al., 2011; Powell et al., 2005; Taylor & Dando, 2018). Indeed, the social cognition literature concerning memory performance when social demand is controlled offers several potential explanations for our pattern of results (e.g., Vredeveldt et al., 2011; Wagstaff et al., 2011). Physical co-presence can impose dual task demands, reducing resources available for effortful remembering (Koutstaal et al., 2001; Vredeveldt et al., 2018; Vredeveldt et al., 2011). Monitoring of social, physical and linguistic cues while simultaneously recalling episodic information has been found to negatively impact retrieval and control processes Koriat & Goldsmith, 1994, 1996; Shapira & Pansky, 2019).

In a VE, dual task demands may be reduced since parties are not physically co-present. Rather, each is represented by an avatar, and they communicate as such. Hence, the social environment may be less demanding. Nonetheless, VEs allow an immersive experience, supporting natural behaviours and effective communication (Lee & Marsella, 2006; Shardaet al., 2006). Our feedback reveals participants interviewed in the VE reported reduced social demands, potentially allowing improved cognitive control. Cognitive control supports top-down resource allocation to goal-relevant tasks, here conscious recall (e.g., Braver et al., 2007; Kiyonaga et al., 2012; Savine et al., 2010), which has been found to enhance performance in cognitively complex tasks such as episodic retrieval (e.g., Botvinick et al., 2001; Greene et al., 2004; Hammond & Summers, 1972; Rondeel et al., 2015). Indeed, despite differences in recall duration, that there was parity across conditions for the number of probing (TED) questions asked adds weight to the importance of environment for supporting cognitive effort and the benefits of doing so for improved performance.

Equally, the VE might have promoted memory performance by potentially reducing feelings of anxiety often associated with traditional in-person face-to-face interactions (Davis & Bottoms, 2002; Fisher & Geiselman, 1992). Theories suggest that anxiety can compete for cognitive resources (e.g., Eysenck & Calvo, 1992; Eysenck et al., 2007). Reduced anxiety at retrieval releases cognitive resources for memory search with potential for improving memory output. In this study, VE participants reported feeling more comfortable during the interview compared to the FtF participants, which suggests a more relaxed experience, perhaps. Nevertheless, neither actual nor perceived anxiety was directly measured, and so future research could investigate anxiety and trauma responses in remote interviewing settings.

In line with predictions on the benefits of rapport for leveraging cooperation and eliciting information (e.g., Abbe & Brandon, 2014; K. Collins & Carthy, 2019; Gabbert et al., 2020; Nahouli et al., 2021), our cluster of rapport behaviours were well received and resulted in quantifiable benefits (more information & fewer errors), providing support for H^2^. Where rapport was present in the free recall, fewer errors were made, and more correct information items were reported. In follow-on probing (TED) questioning, additional correct information was reported without concomitant increases in errors. Questioning is vital for gathering additional fine-grained information (Ministry of Justice, 2011). However, this additional information is often accompanied by errors, a consistently reported pattern in adults and children (Dando, 2013; Dando et al., 2009; Köhnken et al., 1999; Mattison et al., 2015; Memon et al., 2010; Milne & Bull, 2002; Milne et al., 2019) and so understanding which types of interviewing technique or interviewer behaviours have potential to mitigate errors is important.

Two possible explanations emerge for why rapport-building might mitigate errors during probing (TED) questioning. First, feedback revealed participants were more comfortable and found it easier to say when they couldn’t remember. This is crucial, since errors increase when witnesses feel pressure (real or perceived) to provide answers even when unsure (Scoboria & Fisico, 2013; see also Ceci& Bruck, 1993). Second, the rapport superiority effect may have carried over from the preceding free-recall phase. Reduced errors from the offset offer some protection as the interview progresses, since interviewees are not then questioned about information initially provided, which unbeknown to them may have been erroneous—where the free-recall is highly accurate, it follows that the questioning phase may be more accurate, also.

The benefit of external task support for improved episodic recall is clear (e.g., Dando et al., 2020; Fisher & Geisleman, 1992; Hope et al., 2014; Mattison et al., 2015; Smith & Vela, 1992). Here, rapport-building behaviours offered support in line with this literature, assuaging the social demands of an interview to support goal-directed allocation of resources (Dando, 2013; Mather & Knight, 2005; Vredeveldt et al., 2018; Vredeveldt et al., 2011; Wagstaff et al., 2011). Saying “I do not know” or “I can’t remember” allows witnesses to withhold information they are less confident about (see Koriat & Goldsmith, 1994). Low confidence responses are typically less accurate than high confidence responses (e.g., Evans & Fisher, 2011; Wixted et al., 2018), although not always (e.g., Berkowitz et al., 2020; Sauer et al., 2019). Feedback revealed participants in the rapport condition were more confident in their memory and were more comfortable saying they did not know/could not remember.

Finally, participants in the VE + rapport condition outperformed all others. While the standalone benefits of rapport-building and the VE are apparent, our performance and feedback results indicate these benefits were additive in this condition. The VE apparently reducing feelings of anxiety associated with traditional in-person FtF interactions alongside rapport-building behaviours, which feedback indicated had lowered the cognitive task demands. We did not, however, observe a similar pattern of interaction results in the FtF + rapport condition, thereby adding to the literature concerning the potential the benefits of VEs as interviewing spaces (e.g., Mousas et al., 2018; Saarijärvi & Bratt, 2021; Sutherland, 2020) and the impact of environment on complex cognition. The locus of improved performance in the VE + rapport condition was the free recall, where 20% to 35% more correct information was reported than in the rapport-absent face-to-face and rapport-absent VE conditions.

Correct recall interactions were nonsignificant in the follow-on questioning phase. In contrast, errors were high in face-to-face rapport-absent interviews, an increase of over 100% compared to the VE rapport-absent condition. The importance of rapport irrespective of environment is clear, but in face-to-face contexts rapport appears particularly important for mitigating errors and increasing information gain. Our findings add to the importance of rapport (e.g., R. Collins et al., 2002; Nahouli et al., 2021; Risan et al., 2016) for interpersonal communication (e.g., Abbe & Brandon, 2014; Alison et al., 2013; Gabbert et al., 2021) and offer novel insights into the use of rapport in VEs for investigative purposes. As reported elsewhere, we too found rapport could be built between agents in VE and that agent-generated rapport-building was effective (e.g., Gratch et al., 2006; Hale & Antonia, 2016; Herrera et al., 2020; Rotman & Wu, 2015).

Limitations should be noted. Mock witness paradigms do not precisely replicate the experiences of real eyewitnesses. Nonetheless, some social and cognitive demands were present. Participants were recruited from the general population and were unfamiliar with eyewitness research but were made aware that memory was important and would be assessed. This demand characteristic is present with real witnesses who understand the importance of their memory performance (Fisher et al., 2017; Geiselman & Fisher, 2014; Hoogesteyn et al., 2020) and the need to provide detailed information. Further limitations stem from our operationalisation of a series of basic techniques, thus reducing a multifaceted social behaviour to individual components. It is likely that the value of rapport is far more. A priori power analysis (Faul et al., 2007) revealed our sample size was more than adequate to detect large effects but would not be powerful enough to detect small effects. Future research might consider larger sample sizes towards a more nuanced understanding, although the impact of small effect sizes for applied research is currently the subject of discussion (see Götz et al., 2022; Primbs et al., 2022). We did not consider the impact of individual rapport behaviours and neither did we collect formal interviewer feedback in terms of perceived challenges and benefits of interviewing in a VE, for example.

Despite limitations common to most applied research of this nature, our findings advance understanding of the positive impact of basic rapport-building behaviours *per se*, and as far as we are aware is the first to have highlighted the impact of rapport-building in avatar-to-avatar investigative witness interview contexts. Virtual reality technologies have significant, yet-to-be-fully realised potential to change and improve professional practice in terms of apparently seamlessly supporting prosocial compliance and improving associated cognitions. Increased availability of VR headsets allows people to easily communicate in VEs using accessible platforms. Most participants (+80%) had never used VR headsets but reported them easy to use and were open to being interviewed in VEs. However, availability and accessibility of hardware and software and end-user acceptability requires further attention, although pre COVID-19 government statistics reveal 91% of UK adults already use web-based platforms (Office of National Statistics, 2019). Since the COVID-19 pandemic, digital adoption has taken a quantum leap, changing the way that organisations do business, including police and government bodies (e.g., national crime agency) concerned with security. Accordingly, cognition in VEs must be further investigated, and research is urgently needed to better understand rapport in a diverse range of investigative contexts.

Nonetheless, this research does provide much needed insight into the importance of task support for complex cognition in applied settings in terms of considering both retrieval environment and managing prosocial behaviours. Our results illuminate the importance of a cluster of basic prosocial behaviours used in combination, and as such offer interviewing professionals additional “tools” towards improved outcomes, and a way of practicing and honing their rapport-building and interviewing skills which seem very likely to port across to more traditional in-person face-to-face contexts.

## Footnotes

1 From here on we use the term witness to refer to witnesses, victims and survivors of crime.

## References

[CR1] Abbe A, Brandon SE (2014). Building and maintaining rapport in investigative interviews. Police Practice and Research.

[CR2] Ahn SJ, Le AMT, Bailenson J (2013). The effect of embodied experiences on self-other merging, attitude, and helping behavior. Media Psychology.

[CR3] Alison LJ, Alison E, Noone G, Elntib S, Christiansen P (2013). Why tough tactics fail and rapport gets results: Observing Rapport-Based Interpersonal Techniques (ORBIT) to generate useful information from terrorists. Psychology, Public Policy, and Law.

[CR4] Baccon LA, Chiarovano E, MacDougall HG (2019). Virtual reality for teletherapy: Avatars may combine the benefits of face-to-face communication with the anonymity of online text-based communication. Cyberpsychology, Behavior, and Social Networking.

[CR5] Baker-Eck B, Bull R, Walsh D (2020). Investigative empathy: A strength scale of empathy based on European police perspectives. Psychiatry, Psychology and Law.

[CR6] Bailenson JN, Davies A, Blascovich J, Beall AC, McCall C, Guadagno RE (2008). The effects of witness viewpoint distance, angle, and choice on eyewitness accuracy in police lineups conducted in immersive virtual environments. Presence: Teleoperators and Virtual Environments.

[CR7] Bente G, Rüggenberg S, Krämer NC, Eschenburg F (2008). Avatar-mediated networking: Increasing social presence and interpersonal trust in net-based collaborations. Human Communication Research.

[CR8] Bethel, C. L., Eakin, D., Anreddy, S., Stuart, J. K., & Carruth, D. (2013, March). Eyewitnesses are misled by human but not robot interviewers. In *2013 8*^*th*^*ACM/IEEE International Conference on Human-Robot Interaction (HRI)* (pp. 25–32). IEEE.

[CR9] Botvinick MM, Braver TS, Barch DM, Carter CS, Cohen JD (2001). Conflict monitoring and cognitive control. Psychological Review.

[CR10] Braver TS, Gray JR, Burgess GC (2007). Explaining the many varieties of working memory variation: Dual mechanisms of cognitive control. Variation in Working Memory.

[CR11] Ceci SJ, Bruck M (1993). Suggestibility of the child witness: a historical review and synthesis. Psychological Bulletin.

[CR12] Berkowitz, S. R., Garrett, B. L., Fenn, K. M., & Loftus, E. F. (2020). Convicting with confidence? Why we should not over-rely on eyewitness confidence. *Memory*, 1–6. 10.1080/09658211.2020.1849308 33228497

[CR13] Brewer MB, Feinstein ASH (1999). Dual processes in the cognitive representation of persons and social categories.

[CR14] College of Policing (2018) Investigative interviewing. https://www.college.police.uk/guidance/obtaining-initial-accounts/rapportbuilding

[CR15] College of Policing (2021) Rapport building. https://www.college.police.uk/guidance/obtaining-initial-accounts/rapport-building

[CR16] Collins R, Lincoln R, Frank MG (2002). The effect of rapport in forensic interviewing. Psychiatry, Psychology and Law.

[CR17] Collins K, Carthy N (2019). No rapport, no comment: The relationship between rapport and communication during investigative interviews with suspects. Journal of Investigative Psychology and Offender Profiling.

[CR18] Conway MA, Pleydell-Pearce CW (2000). The construction of autobiographical memories in the self-memory system. Psychological Review.

[CR19] Dando CJ (2013). Drawing to remember: External support of older adults’ eyewitness performance. PLOS ONE.

[CR20] Dando, C. J., & Oxburgh, G. E. (2016). Empathy in the field: towards a taxonomy of empathic communication in information gathering interviews with suspected sex offenders1 2 3. *European Journal of Psychology Applied to Legal Context* (Internet), 27–33.

[CR21] Dando CJ, Ormerod TC (2020). Noncoercive human intelligence gathering. Journal of Experimental Psychology: General.

[CR22] Dando C, Wilcock R, Milne R, Henry L (2009). A modified cognitive interview procedure for frontline police investigators. Applied Cognitive Psychology: The Official Journal of the Society for Applied Research in Memory and Cognition.

[CR23] Dando CJ, Geiselman RE, MacLeod N, Griffiths A, Oxburgh G, Myklebust T, Grant T, Milne R (2016). Interviewing adult witnesses and victims. *Communication in investigative and legal contexts: Integrated approaches from forensic psychology, linguistics and law enforcement*.

[CR24] Davis SL, Bottoms BL (2002). Effects of social support on children’s eyewitness reports: A test of the underlying mechanism. Law and Human Behavior.

[CR25] Drolet AL, Morris MW (2000). Rapport in conflict resolution: Accounting for how face-to-face contact fosters mutual cooperation in mixed-motive conflicts. Journal of Experimental Social Psychology.

[CR26] Evans JR, Fisher RP (2011). Eyewitness memory: Balancing the accuracy, precision and quantity of information through metacognitive monitoring and control. Applied Cognitive Psychology.

[CR27] Eysenck MW, Calvo MG (1992). Anxiety and performance: The processing efficiency theory. Cognition and Emotion.

[CR28] Eysenck MW, Derakshan N, Santos R, Calvo MG (2007). Anxiety and cognitive performance: Attentional control theory. Emotion.

[CR29] Fahsing I, Rachlew A, Williamson T, Milne R, Savage SP (2009). Investigative interviewing in the Nordic region. *International developments in investigative interviewing*.

[CR30] Fängström K, Salari R, Eriksson M, Sarkadi A (2017). The computer-assisted interview In My Shoes can benefit shy preschool children’s communication. PLOS ONE.

[CR31] Faul F, Erdfelder E, Lang AG, Buchner A (2007). G* Power 3: A flexible statistical power analysis program for the social, behavioral, and biomedical sciences. Behavior Research Methods.

[CR32] Fisher RP, Geiselman RE (1992). Memory enhancing techniques for investigative interviewing: The cognitive interview.

[CR33] Fisher RP, Geiselman RE (1992). *Memory enhancing techniques for investigative interviewing: The cognitive interview*.

[CR34] Frith CD, Frith U (2012). Mechanisms of social cognition. Annual Review of Psychology.

[CR35] Fiske ST, Taylor SE (2013). *Social cognition: From brains to culture*.

[CR36] Fisher RP, Milne R, Bull R (2011). Interviewing cooperative witnesses. Current Directions in Psychological Science.

[CR37] Fisher RP, Ross SJ, Cahill BS (2017). Interviewing witnesses and victims. *Forensic psychology in context*.

[CR38] Fiske, S. T., Lin, M., & Neuberg, S. L. (2018). The continuum model: Ten years later. *Social Cognition*, 41–75.

[CR39] Gabbert F, Hope L, Luther K, Wright G, Ng M, Oxburgh G (2020). Rapport systematic review.

[CR40] Gabbert F, Hope L, Luther K, Wright G, Ng M, Oxburgh G (2021). Exploring the use of rapport in professional information-gathering contexts by systematically mapping the evidence base. Applied Cognitive Psychology.

[CR41] Gallese V, Keysers C, Rizzolatti G (2004). A unifying view of the basis of social cognition. Trends in Cognitive Sciences.

[CR42] Geiselman RE, Fisher RP (2014). Interviewing witnesses and victims. *Investigative interviewing: The essentials*.

[CR43] Gonzalez-Franco M, Lanier J (2017). Model of illusions and virtual reality. Frontiers in Psychology.

[CR44] Götz FM, Gosling SD, Rentfrow PJ (2022). Small effects: The indispensable foundation for a cumulative psychological science. Perspectives on Psychological Science.

[CR45] Gratch, J., Okhmatovskaia, A., Lamothe, F., Marsella, S., Morales, M., van der Werf, R. J., & Morency, L. P. (2006). Virtual rapport. In *International Workshop on Intelligent Virtual Agents* (pp. 14–27). Springer.

[CR46] Greene JD, Nystrom LE, Engell AD, Darley JM, Cohen JD (2004). The neural bases of cognitive conflict and control in moral judgment. Neuron.

[CR47] Greiner B, Caravella M, Roth AE (2014). Is avatar-to-avatar communication as effective as face-to-face communication? An Ultimatum Game experiment in First and Second life. Journal of Economic Behavior & Organization.

[CR48] Griffiths A, Rachlew A (2018). From interrogation to investigative interviewing: The application of psychology. *The psychology of criminal investigation*.

[CR49] Haginoya S, Yamamoto S, Santtila P (2021). The combination of feedback and modelling in online simulation training of child sexual abuse interviews improves interview quality in clinical psychologists. Child Abuse & Neglect.

[CR50] Hale J, Antonia FDC (2016). Testing the relationship between mimicry, trust and rapport in virtual reality conversations. Scientific Reports.

[CR51] Hamilton G, Whiting EA, Brubacher SP, Powell MB (2017). The effects of face-to-face versus live video-feed interviewing on children’s event reports. Legal and Criminological Psychology.

[CR52] Hammond KR, Summers DA (1972). Cognitive control. Psychological review.

[CR53] Herrera F, Bailenson J, Weisz E, Ogle E, Zaki J (2018). Building long-term empathy: A large-scale comparison of traditional and virtual reality perspective-taking. PloS One.

[CR54] Herrera F, Oh SY, Bailenson JN (2020). Effect of behavioral realism on social interactions inside collaborative virtual environments. Presence.

[CR55] Holmberg U, Madsen K (2014). Rapport operationalized as a humanitarian interview in investigative interview settings. Psychiatry, Psychology and Law.

[CR56] Hoogesteyn K, Meijer E, Vrij A (2020). Examining witness interviewing environments. Journal of Investigative Psychology and Offender Profiling.

[CR57] Hope L, Gabbert F, Fisher RP (2011). From laboratory to the street: Capturing witness memory using the Self-Administered Interview. Legal and Criminological Psychology.

[CR58] Jakobsen KK (2021). Empathy in investigative interviews of victims: How to understand it, how to measure it, and how to do it?. Police Practice and Research.

[CR59] Joinson AN (2001). Self-disclosure in computer-mediated communication: The role of self-awareness and visual anonymity. European journal of Social Psychology.

[CR60] Kieckhaefer JM, Vallano JP, Schreiber Compo N (2014). Examining the positive effects of rapport-building: When and why does rapport-building benefit adult eyewitness memory?. Memory.

[CR61] Kang SH, Watt JH (2013). The impact of avatar realism and anonymity on effective communication via mobile devices. Computers in Human Behavior.

[CR62] Kiyonaga A, Egner T, Soto D (2012). Cognitive control over working memory biases of selection. Psychonomic Bulletin & Review.

[CR63] Köhnken G, Milne R, Memon A, Bull R (1999). The cognitive interview: A meta-analysis. Psychology, Crime and Law.

[CR64] Kontogianni F, Hope L, Taylor PJ, Vrij A, Gabbert F (2020). “Tell me more about this…”: An examination of the efficacy of follow-up open questions following an initial account. Applied Cognitive Psychology.

[CR65] Koriat A, Goldsmith M (1994). Memory in naturalistic and laboratory contexts: Distinguishing the accuracy-oriented and quantity-oriented approaches to memory assessment. Journal of Experimental Psychology: General.

[CR66] Koriat A, Goldsmith M (1996). Monitoring and control processes in the strategic regulation of memory accuracy. Psychological Review.

[CR67] Koutstaal W, Schacter DL, Brenner C (2001). Dual task demands and gist-based false recognition of pictures in younger and older adults. Journal of Memory and Language.

[CR68] Kuivaniemi-Smith HJ, Nash RA, Brodie ER, Mahoney G, Rynn C (2014). Producing facial composite sketches in remote Cognitive Interviews: A preliminary investigation. Psychology, Crime & Law.

[CR69] Lee, J. J., & Dryjanska, L. (2019). Avatars and digital technology literacy applied in psychology. *Handbook of research on media literacy research and applications across disciplines* (pp. 402–420). IGI Global.

[CR70] Lee, J., & Marsella, S. (2006) Nonverbal behavior generator for embodied conversational agents. *International Workshop on Intelligent Virtual Agents* (pp. 243–255). Springer.

[CR71] Loomis JM, Blascovich JJ, Beall AC (1999). Immersive virtual environment technology as a basic research tool in psychology. Behavior Research Methods, Instruments, & Computers.

[CR72] Maddox KB, Rapp DN, Brion S, Taylor HA (2008). Social influences on spatial memory. Memory & Cognition.

[CR73] Macrae CN, Bodenhausen GV (2000). Social cognition: Thinking categorically. Annual Review of Psychology.

[CR74] Mattison ML, Dando CJ, Ormerod TC (2015). Sketching to remember: Episodic free-recall task support for child witnesses and victims with autism spectrum disorder. Journal of Autism and Developmental Disorders.

[CR75] Mather M, Knight M (2005). Goal-directed memory: The role of cognitive control in older adults’ emotional memory. Psychology and Aging.

[CR76] Meissner CA, Kelly CE, Woestehoff SA (2015). Improving the effectiveness of suspect interrogations. Annual Review of Law and Social Science.

[CR77] Memon A, Higham PA (1999). A review of the cognitive interview. Psychology, Crime and Law.

[CR78] Memon A, Meissner CA, Fraser J (2010). The Cognitive Interview: A meta-analytic review and study space analysis of the past 25 years. Psychology, Public Policy, and Law.

[CR79] Milne B, Bull R (1999). Investigative interviewing: Psychology and practice.

[CR80] Milne R, Bull R (2016). Witness interviews and crime investigation. In *An introduction to applied cognitive psychology*.

[CR81] Milne R, Bull R (2002). Back to basics: A componential analysis of the original cognitive interview mnemonics with three age groups. Applied Cognitive Psychology: The Official Journal of the Society for Applied Research in Memory and Cognition.

[CR82] Milne B, Griffiths A, Clarke C, Dando C (2019). The cognitive interview: A tiered approach in the real world. In *Evidence-Based Investigative Interviewing*.

[CR83] Ministry of Justice. (2011). *Achieving best evidence*. Author.

[CR84] Ministry of Justice (2022). Achieving best evidence in criminal proceedings.

[CR85] Mousas C, Anastasiou D, Spantidi O (2018). The effects of appearance and motion of virtual characters on emotional reactivity. Computers in Human Behavior.

[CR86] Nahouli Z, Dando CJ, Mackenzie JM, Aresti A (2021). Rapport-building and witness memory: Actions may ‘speak’ louder than words. PLOS ONE.

[CR87] Nash RA, Houston KA, Ryan K, Woodger N (2014). Remembering remotely: would video-mediation impair witnesses' memory reports?. Psychology, Crime & Law.

[CR88] Nash RA, Nash A, Morris A, Smith SL (2016). Does rapport-building boost the eyewitness eyeclosure effect in closed questioning?. Legal and Criminological Psychology.

[CR89] Nash A, Ridout N, Nash RA (2020). Facing away from the interviewer: Evidence of little benefit to eyewitnesses' memory performance. Applied Cognitive Psychology.

[CR90] Novotny E, Frank MG, Grizzard M (2021). A laboratory study comparing the effectiveness of verbal and nonverbal rapport-building techniques in interviews. Communication Studies.

[CR91] Nunan, J., Stanier, I., Milne, R., Shawyer, A., Walsh, D., & May, B. (2020). The impact of rapport on intelligence yield: Police source handler telephone interactions with covert human intelligence sources. *Psychiatry, Psychology and Law*, 1–19. 10.1080/13218719.2020.1784807 PMC9186345 35693385

[CR92] Office of National Statics. (2019). *Home internet and social media usage.* https://www.ons.gov.uk/peoplepopulationandcommunity/householdcharacteristics/homeinternetandsocialmediausage

[CR93] Omarzu J (2000). A disclosure decision model: Determining how and when individuals will self-disclose. Personality and Social Psychology Review.

[CR94] Oxburgh, G. E., Myklebust, T., & Grant, T. (2010). The question of question types in police interviews: A review of the literature from a psychological and linguistic perspective. *International Journal of Speech, Language & the Law, 17*(1).

[CR95] Peyroux E, Franck N (2014). RC2S: A cognitive remediation program to improve social cognition in schizophrenia and related disorders. Frontiers in Human Neuroscience.

[CR96] Pickard MD, Roster CA, Chen Y (2016). Revealing sensitive information in personal interviews: Is self-disclosure easier with humans or avatars and under what conditions?. Computers in Human Behavior.

[CR97] Pompedda, F., Zhang, Y., Haginoya, S., & Santtila, P. (2022). A mega-analysis of the effects of feedback on the quality of simulated child sexual abuse interviews with avatars. *Journal of Police and Criminal Psychology*, 1–14.

[CR98] Powell, M. B., Fisher, R. P., & Wright, R. (2005). Investigative interviewing. *Psychology and law: An empirical perspective* (pp. 11–42).

[CR99] Primbs, M. A., Pennington, C. R., Lakens, D., Silan, M. A. A., Lieck, D. S., Forscher, P. S., ... Westwood, S. J. (2022). Are small effects the indispensable foundation for a cumulative psychological science? A reply to Götz et al. (2022). *Perspectives on Psychological Science*. 10.1177/17456916221100420 PMC10018048 36126652

[CR100] Rachlew A, Fahsing I, Aarli R, Hedlund M-A, Jebens SE (2015). Politiavhøret [The investigative interview]. Bevis i straffesaker [Evidence in criminal proceedings].

[CR101] Rehm IC, Foenander E, Wallace K, Abbott JAM, Kyrios M, Thomas N (2016). What role can avatars play in emental health interventions? Exploring new models of client–therapist interaction. Frontiers in Psychiatry.

[CR102] Risan P, Binder PE, Milne R (2016). Regulating and coping with distress during police interviews of traumatized victims. Psychological Trauma: Theory, Research, Practice, and Policy.

[CR103] Roth, D., Waldow, K., Latoschik, M. E., Fuhrmann, A., & Bente, G. (2017). Socially immersive avatar-based communication. In *2017 IEEE Virtual Reality (VR)* (pp. 259–260). IEEE.

[CR104] Roberts KP, Lamb ME, Sternberg KJ (2004). The effects of rapport-building style on children’s reports of a staged event. Applied Cognitive Psychology.

[CR105] Rondeel E, Van Steenbergen H, Holland R, van Knippenberg A (2015). A closer look at cognitive control: Differences in resource allocation during updating, inhibition and switching as revealed by pupillometry. Frontiers in Human Neuroscience.

[CR106] Rotman, D., & Wu, P. F. (2015). Sense of community in virtual environments. In *Virtual communities: 2014* (pp. 50–62). Routledge.

[CR107] Rubin Z (1975). Disclosing oneself to a stranger: Reciprocity and its limits. Journal of Experimental Social Psychology.

[CR108] Saarijärvi M, Bratt EL (2021). When face-to-face interviews are not possible: Tips and tricks for video, telephone, online chat, and email interviews in qualitative research. European Journal of Cardiovascular Nursing.

[CR109] Saidel-Goley IN, Albiero EE, Flannery KA (2012). An evaluation of nonclinical dissociation utilizing a virtual environment shows enhanced working memory and attention. Cyberpsychology, Behavior, and Social Networking.

[CR110] Salmon G, Nie M, Edirisingha P (2010). Developing a five-stage model of learning in Second Life. Educational Research.

[CR111] Sauer JD, Palmer MA, Brewer N (2019). Pitfalls in using eyewitness confidence to diagnose the accuracy of an individual identification decision. Psychology, Public Policy, and Law.

[CR112] Sauerland M, Brackmann N, Otgaar H (2018). Rapport: Little effect on children’s, adolescents’, and adults’ statement quantity, accuracy, and suggestibility. Journal of Child Custody.

[CR113] Savine AC, Beck SM, Edwards BG, Chiew KS, Braver TS (2010). Enhancement of cognitive control by approach and avoidance motivational states. Cognition and Emotion.

[CR114] Scoboria A, Fisico S (2013). Encouraging and clarifying “don't know” responses enhances interview quality. Journal of Experimental Psychology: Applied.

[CR115] Segal, A., Pompedda, F., Haginoya, S., Kaniušonytė, G., & Santtila, P. (2022). Avatars with child sexual abuse (vs. no abuse) scenarios elicit different emotional reactions. *Psychology, Crime & Law*, 1–21.

[CR116] Shapira AA, Pansky A (2019). Cognitive and metacognitive determinants of eyewitness memory accuracy over time. Metacognition and Learning.

[CR117] Suler J (2004). The online disinhibition effect. Cyberpsychology & Behavior.

[CR118] Sun, H. (2014). *Rapport and its impact on the disclosure of sensitive information in standardized interviews* (Doctoral dissertation, University of Maryland, College Park).

[CR119] Sutherland LA (2020). The ‘desk-chair countryside’: Affect, authenticity and the rural idyll in a farming computer game. Journal of Rural Studies.

[CR120] Sharda R, Delen D (2006). Predicting box-office success of motion pictures with neural networks. Expert Systems with Applications.

[CR121] Slater M (2009). Place illusion and plausibility can lead to realistic behaviour in immersive virtual environments. Philosophical Transactions of the Royal Society B: Biological Sciences.

[CR122] Smith SM, Vela E (1992). Environmental context-dependent eyewitness recognition. Applied Cognitive Psychology.

[CR123] Taylor DA, Dando CJ (2018). Eyewitness memory in face-to-face and immersive avatar-to-avatar contexts. Frontiers in Psychology.

[CR124] Tulving E (1993). What is episodic memory?. Current Directions in Psychological Science.

[CR125] Vallano JP, Compo NS (2011). A comfortable witness is a good witness: Rapport-building and susceptibility to misinformation in an investigative mock-crime interview. Applied Cognitive Psychology.

[CR126] Vallano JP, Schreiber Compo N (2011). A comfortable witness is a good witness: Rapport-building and susceptibility to misinformation in an investigative mock-crime interview. Applied Cognitive Psychology.

[CR127] Vredeveldt A, Charman SD, den Blanken A, Hooydonk M (2018). Effects of cannabis on eyewitness memory: A field study. Applied Cognitive Psychology.

[CR128] Vredeveldt A, Hitch GJ, Baddeley AD (2011). Eyeclosure helps memory by reducing cognitive load and enhancing visualisation. Memory & cognition.

[CR129] Vrij A, Hope L, Fisher RP (2014). Eliciting reliable information in investigative interviews. Policy Insights from the Behavioral and Brain Sciences.

[CR130] Walsh D, Bull R (2012). Examining rapport in investigative interviews with suspects: Does its building and maintenance work?. Journal of Police and Criminal Psychology.

[CR131] Webster WS, Oxburgh GE, Dando CJ (2021). The use and efficacy of question type and an attentive interviewing style in adult rape interviews. Psychology, Crime & Law.

[CR132] Witmer BG, Singer MJ (1998). Measuring presence in virtual environments: A presence questionnaire. Presence.

[CR133] Wagstaff GF, Wheatcroft JM, Caddick AM, Kirby LJ, Lamont E (2011). Enhancing witness memory with techniques derived from hypnotic investigative interviewing: Focused meditation, eye-closure, and context reinstatement. International Journal of Clinical and Experimental Hypnosis.

[CR134] Wixted JT, Mickes L, Fisher RP (2018). Rethinking the reliability of eyewitness memory. Perspectives on Psychological Science.

